# Sandwich-type double-layer piezoelectric nanogenerators based on one- and two-dimensional ZnO nanostructures with improved output performance

**DOI:** 10.1038/s41598-023-43047-4

**Published:** 2023-09-29

**Authors:** Parisa Fakhri, Naeimeh Eaianli, Roohollah Bagherzadeh, Babak Jaleh, Mohammad Kashfi, Rui Fausto

**Affiliations:** 1 Instrumentation Research Group, Niroo Research Institute (NRI), Tehran, Iran; 2 Department of Physics, Faculty of Science, Bu-Ali Sina University, Hamedan, Iran; 3 Institute for Advanced Textile Materials and Technologies, Textile Engineering Department, Amirkabir University of Technology, Tehran, Iran; 4 Mechanical Engineering Department, Ayatollah Boroujerdi University, Boroujerd, Iran; 5 CQC-IMS, Department of Chemistry, University of Coimbra, 3004-525 Coimbra, Portugal; 6 Faculty of Sciences and Letters, Department of Physics, Istanbul Kultur University, Ataköy Campus, Bakirköy, 34156 Istanbul, Turkey

**Keywords:** Energy science and technology, Engineering, Materials science, Nanoscience and technology

## Abstract

Piezoelectric nanogenerators (PENGs) have attracted great interest owing to their broad range application in environmental mechanical energy harvesting to power small electronic devices. In this study, novel flexible and high-performance double-layer sandwich-type PENGs based on one-dimensional (1-D) and two-dimensional (2-D) zinc oxide (ZnO) nanostructures and Ni foam as the middle layer have been developed. The morphology and structure of 1- and 2-D ZnO nanostructures have been studied by scanning electron microscopy (SEM) and X-ray diffraction (XRD). To investigate the effect of structural design on the piezoelectric performance, single-layer PENGs were also fabricated. The piezoelectric output of all prepared PENGs were evaluated under different human impacts at various forces and frequencies. The double-layer designed PENGs showed a two times larger voltage output compared to the single-layer PENGs, and the use of Ni foam as middle-layer and of 2-D ZnO nanosheets (compared to 1-D nanorods) was also found to increase the performance of the designed PENGs. The working mechanism of the prepared PENGs is also discussed. The design of nanogenerators as double-layer sandwich structures instead of two integrated single-layer devices reduces the overall preparation time and processing steps and enhances their output performance, thus opening the gate for widening their practical applications.

## Introduction

Portable electronic devices have become an integral part of our daily life and play an important role in several domains of activity, such as health care, implantable medical systems, communication, and environmental monitoring^1,2^. The growing demand, along with the development of portable and wearable systems in recent years, led to an increased interest in the development of renewable and independent energy sources for powering those devices^3^. Thereby, self-powered technologies based on energy harvesting or in situ generated charges from environmental sources, such as solar, mechanical, and thermal, have garnered a lot of attention^4–6^.

Harvesting thermal and mechanical energies from the ambient and converting them into electrical energy, nanogenerators have been introduced to meet the need for self-powered devices^6,7^. Nanogenerators are mainly based on three effects: piezoelectric and triboelectric effects, for mechanical energy harvesting, and the pyroelectric effect, for thermal energy harvesting^8,9^. Owing to the abundant availability of ambient mechanical energy sources, such as waves, wind, vibrations, human motion, etc., mechanical energy harvesting is a qualified candidate for the production of electricity^10–12^.

The first piezoelectric nanogenerator based on ZnO was introduced in 2006^13^. With the rapid growth of the energy harvesting technology, various piezoelectric nanogenerator (PENG) devices based on piezoelectric materials, such as lead zirconate titanate (Pb[Zr_x_Ti_1-x_]O_3_ with 0 ≤ x ≤ 1; abbreviated as PZT)^14^, polyvinylidene difluoride ((C_2_H_2_F_2_)n; PVDF) and barium titanate (BaTiO_3_)^15^, have been described. Among the various piezoelectric materials, ZnO has been widely studied due to its unique piezoelectric, semiconducting, optical and surface properties^16–20^. Moreover, ZnO can be easily synthesized in different nanostructures using a variety of synthesis techniques^21^.

Many investigations on PENGs have been focused on the enhancement of their output power, which is one of the main challenges in the development of piezoelectric devices suitable for practical uses^22,23^. In particular, several approaches have been developed for improving the output performance of ZnO-based PENGs^21^. Recently, some studies have demonstrated that double-layered designed ZnO-based PENGs can improve the energy harvesting performance^24,25^. For example, Shin et al.^24^ introduced a double heterostructure of the type (ZnO nanorods)-graphene-(ZnO nanorods), in which the increment of the number of nanorods resulted in the improvement of the piezoelectric output up to two times that of the single heterostructure. In turn, Jung et al. fabricated a PENG based on ZnO nanorods on double-sided stainless steel foil, and found that the voltage output of the designed PENG was larger than the sum of the output voltages obtained from the individual sides of the stainless steel substrate, a result that the authors interpreted as being due to an electric field induced synergetic effect^25^.

The multi-layer design of PENGs including a conductive intermediate layer has been demonstrated to improve the output performance^26–29^. Yoon et al.^27^, developed a sandwich-type ZnO-Ag-ZnO stacked PENG, which was able to generate a relatively high output voltage compared to the single ZnO-layered device. However, they found that the developed device was structurally weak when subjected to mechanical forces, owing to hardening and brittleness of the Ag paste layer over time^29^. Therefore, they developed a new sandwich-type ZnO piezoelectric nanogenerator composed of stacked layers of ZnO-(carbon tape)-ZnO, and demonstrated that the use of a conductive carbon tape leads to significant increase in the output voltage^29^. In turn, Jung and co-workers^28^ incorporated a Cu interlayer between the ZnO thin films to form a multi-layered structure, and showed that the piezoelectric output is enhanced by intervention of a copper conductive layer, in result of the induced interfacial polarization between the ZnO film and the Cu inclusion.

All the sandwich-designed PENGs reported hitherto use ZnO thin films, while ZnO nanostructures, such as nanorods (NRs; 1-D) and nanosheets (NSs; 2-D), owing to their high surface-to-volume ratio, have the potential to improve the properties of the devices. Nevertheless, these types of sandwich-designed PENGs have not been reported yet. This fact stresses the importance of investigating these alternative systems.

In the present work, novel sandwich-type designed flexible PENGs based on one-dimensional (1-D) ZnO nanorods (ZnO NRs) and two-dimensional (2-D) ZnO nanosheets (ZnO NSs), and Ni foam as interlayer, have been developed and their properties evaluated. The Ni foam was used owing to its porous surface, which could be expected to efficiently increase the output voltage of PENGs. The morphology and structure of the ZnO NRs and NSs were characterized by SEM and XRD. The sandwich-type devices were fabricated in a cost-effective way, by inserting the Ni foam between two layers of the vertically grown ZnO nanostructures on flexible substrates. To investigate the effect of the double-layer structure on the output performance of the fabricated devices, the single-layer PENGs were also prepared. The piezoelectric output voltages of all nanogenerators were measured under periodic impacts. The origin of the piezoelectric output improvement of the fabricated multi-layer devices is also briefly addressed.

## Methods

### ZnO nanostructures synthesis

**Figure 1:**
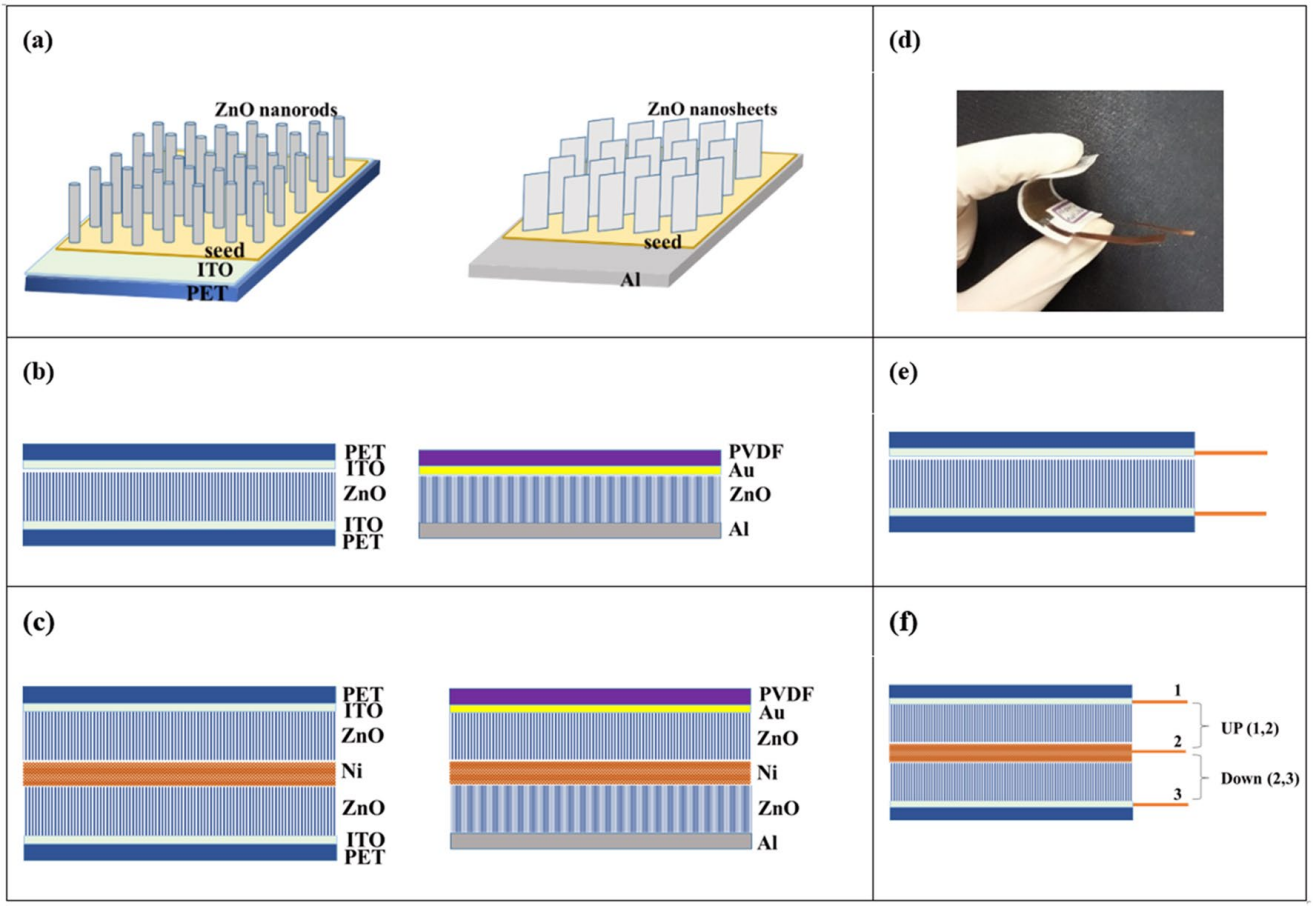
Schematic diagrams of the (**a**) ZnO NRs growth on PET/ITO and ZnO NSs growth on Al substrates, (**b**) single-layer ZnO NRs based S-PENG1 and single-layer ZnO NSs based S-PENG2, (**c**) double-layer ZnO NRs based D-PENG1 and double-layer ZnO NRs and NSs based D-PENG2. (**d**) Real image of flexible D-PENG1. Schematic diagrams of the wire connecting to (**e**) single-layer ZnO NRs based S-PENG1, (**f**) double-layer ZnO NRs based D-PENG1.

The 1- and 2-D ZnO nanostructures were hydrothermally synthesized on seeded substrates according to our previous work^30^. For NRs growth, flexible indium tin coated polyethylene (PET/ITO) was selected as substrate. The substrate was cleaned with acetone, ethanol and deionized water to remove any impurity on the surfaces. It was then deposited by room temperature radio frequency (RF) magnetron sputtering. The sputtering was carried out in an argon atmosphere of 3.5 Pa, at room temperature, and the RF power was 180 W. The seeded substrates were immersed in aqueous solution of zinc nitrate hexahydrate [Zn(NO_3_)_2_·6H_2_O] and hexamethylenetetramine (C_6_H_12_N_4_; HMTA) in deionized water with the mole ratio of 1:1. The growth solution was transferred into a flask and refluxed for 3 h at 90 °C with continuous magnetic stirring. Finally, the substrate was removed from the solution, rinsed with deionized water, and dried at room temperature. Figure 1a shows the schematic diagrams of the ZnO NRs growth on the PET/ITO substrate. For NSs growth, Al foil was selected as substrate and seeded as following. The substrate was coated by 60 mM of zinc acetate in ethanol solution through spin coating and heated at 100 °C. This stage was repeated three times to ensure the uniform formation of the seed layers. Finally, the substrate was placed in an oven for 1 h at 150 °C to form a thick layer of zinc acetate. The growth stage of ZnO nanosheets was carried out using the same method as described for the fabricated NRs. The schematic diagram of the ZnO NSs growth on the Al substrate is shown in Fig. 1a.

### PENGs preparation

The two sets of devices, single-layer PENGs and multi-layer PENGs based on ZnO NRs and NSs, were prepared as schematically shown in Fig. 1b,c. For fabricating the single-layer ZnO NRs based PENG, PET/ITO was used as top electrode. This sample, with the structure PET/ITO-(ZnO NRs)-ITO/PET, was named as S-PENG1. In the case of the single-layer PENG based on ZnO NSs, PVDF/Au was used as top electrode. This nanogenerator, with the structure Al-(ZnO NSs)-Au/PVDF, was designated as S-PENG2.

The double-layer PENGs based on ZnO NRs and NSs were prepared as described below. For multi-layer ZnO NRs based PENG fabrication, a Ni foam was sandwiched between two PET/ITO-(ZnO NRs) layers. The volumetric porosity of the used Ni foam was 90–98%, with a density of 0.15–0.45 g/cm^3^.The double-layer device with the structure PET/ITO-(ZnO NRs)-(Ni foam)-(ZnO NRs)-ITO/PET was named as D-PENG1. For the double-layer PENG based on both ZnO NRs and NSs fabrication, the ZnO NSs lower part of the device was prepared as S-PENG2, while the ZnO NRs were grown on Au as described above for PET/ITO. A Ni foam was sandwiched between the Al-(ZnO NSs) and PVDF/Au-(ZnO NRs) layers. This double-layer sample, with the structure Al-(ZnO NSs)-(Ni foam)-(ZnO NRs)-Au/PVDF, was named as D-PENG2. The dimensions of all devices were 1.5 × 2 cm^2^.

To complete the devices, Cu wires were connected to the electrodes. An image of the prepared D-PENG2 device is shown in Fig. 1d, which illustrates the flexibility of the fabricated PENGs that makes them appropriate choices for wearable electronic devices. For the single-layer PENGs, the Cu wires were connected to the top and bottom electrodes. Figure 1e shows the case of S-PENG1, as example. For the double-layer PENGs, the Cu wires were connected to the top, middle, and bottom electrodes, as shown in Fig. 1f for the case of D-PENG1.

### Characterization

The surface morphologies were investigated by SEM using a XL-30E for ZnO nanostructures and a Tescan MIRA3-XMU for Ni foam. The crystal structures of ZnO was investigated by XRD using an Ital Structures ADP 200 diffractometer in the 2θ range of 30°–60° at the scanning speed of 1.8°/min. The output voltage of the fabricated PENG devices was measured with a digital oscilloscope Agilent Technologies DSO3062A.

## Results

### SEM analyses

**Figure 2:**
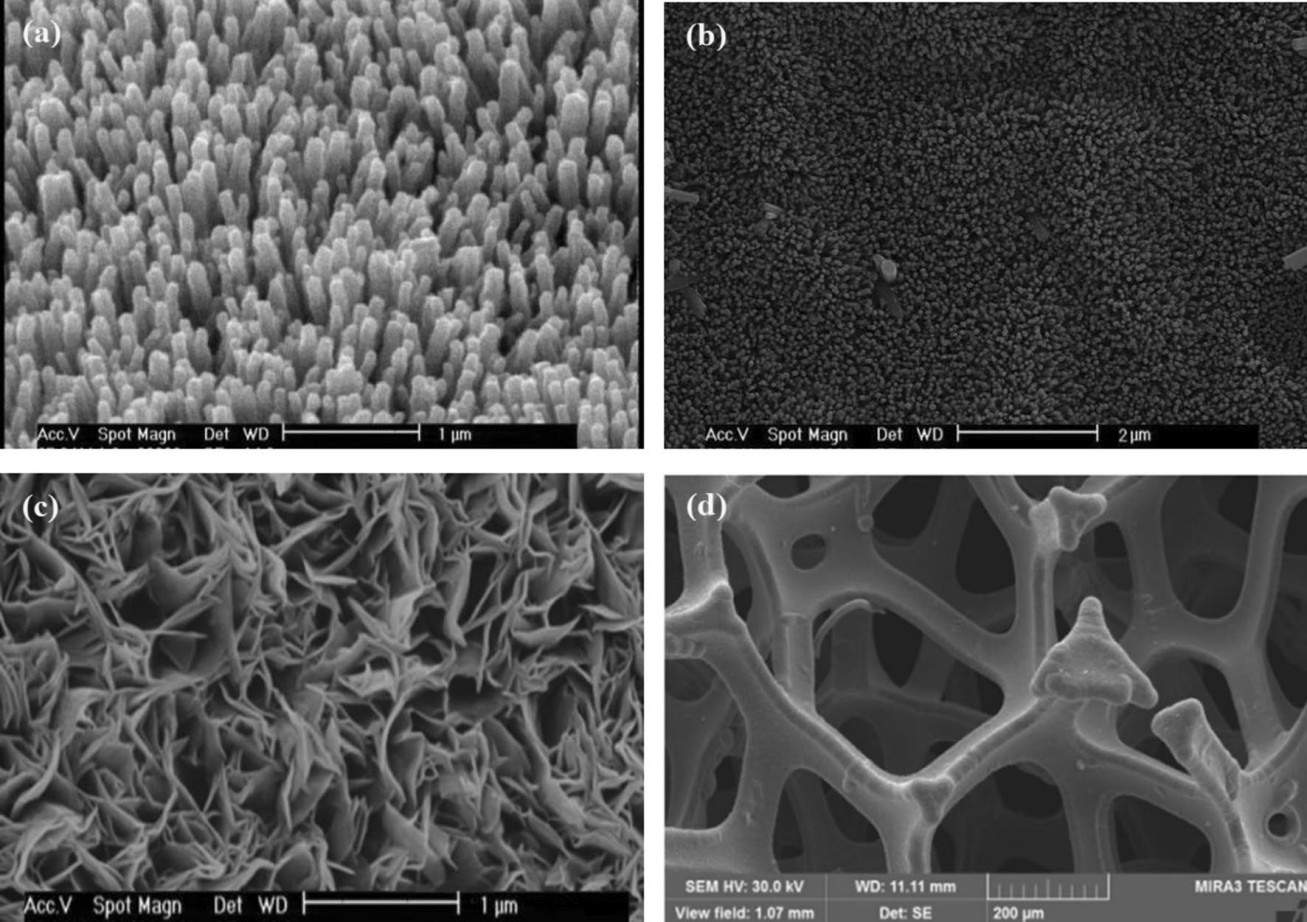
Surface SEM image of (**a**) ZnO NRs on PET/ITO, (**b**) ZnO NRs on PVDF/Au, (**c**) ZnO NSs on Al, and (**d**) Ni foam.

Figure 2a–c shows the SEM images of the obtained ZnO nanostructures on the different substrates used. The SEM images of the surface of ZnO NRs grown on PET/ITO and PVDF/Au are shown in Fig. 2a,b, respectively. As shown in these images, a rather uniform and vertically well-aligned nanorods, with high density, were obtained. The mean diameter of the nanorods grown on ITO and Au substrates were about 90 and 60 nm, respectively. Figure 2c illustrates the SEM image of ZnO NSs grown on Al foil. It can be observed that ZnO NSs are also uniformly and densely grown on the Al substrate, with a thickness of about 50 nm. The SEM image of the Ni foam surface is shown in Fig. 2d.

### XRD analyses

**Figure 3:**
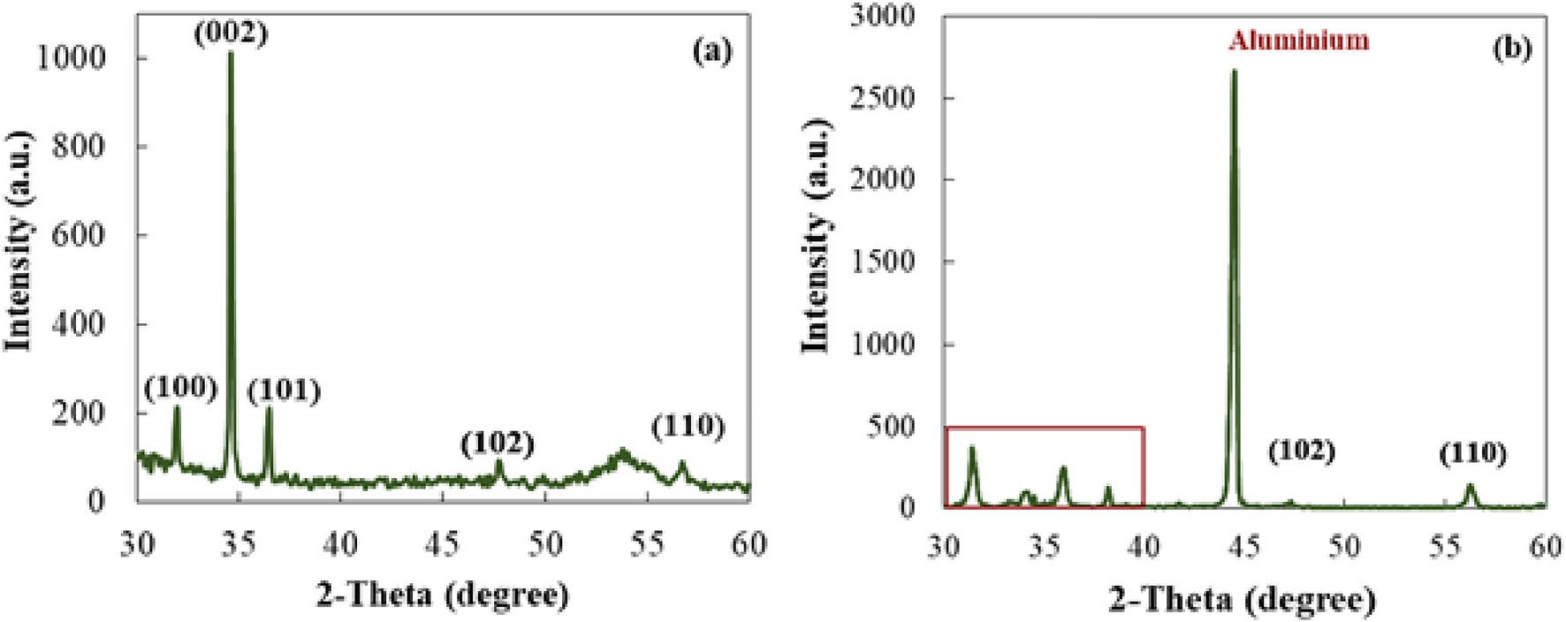
XRD patterns of (**a**) ZnO NRs on PET/ITO and (**b**) ZnO NSs on Al.

The X-ray diffraction analyses were also carried out to further characterize the crystalline structure of the as-grown ZnO nanostructures. The results obtained for ZnO NRs and NSs are illustrated in Fig. 3a,b, respectively. The peaks located at 2θ of 32.3°, 35°, 36.8°, 48.1° and 57° correspond to diffraction from the (100), (002), (101), (102) and (110) planes of the hexagonal structure of ZnO, respectively^31^. The direction of growth of the ZnO NRs grown on PET/ITO could be established by noticing that the intensity of the (002) peak is much higher than those of the remaining peaks (see Fig. 3a), indicating a well-aligned growth of the ZnO NRs along the crystallographic c-axis direction. The XRD pattern of ZnO NRs grown on PVDF/Au was found to be very similar to that of the nanorods grown on PET/ITO. On the other hand, in the case of the ZnO NSs, the XRD data show (100) and (101) peaks with a considerably higher intensity than that of the (002) peak (see Fig. 3b). This outcome shows that the NSs are somewhat tilted and not all grown along the c-axis. The XRD results are fully consistent with the SEM data. The sharp, intense peak at about 44.3° is related with the diffraction from the Al substrate^32^.

### Piezoelectric output

The voltage output of all single- and double-layer prepared nanogenerators has been measured under cyclic impacts. To evaluate the effect of the working frequency on the piezoelectric output of PENGs, an impact experimental setup has been used to trigger PENGs at different frequencies. The employed setup consists of an impactor, a load cell, and an oscilloscope. The samples were placed on the impact stage and the vertical cyclic forces at desired frequencies were applied. The frequencies of the impacts were selected in the range of 1–5 Hz, which is the normal frequency range for the human motions. To ensure repeatability of the results, all experiments were repeated three times, and the median was considered as the response of the device.

**Figure 4:**
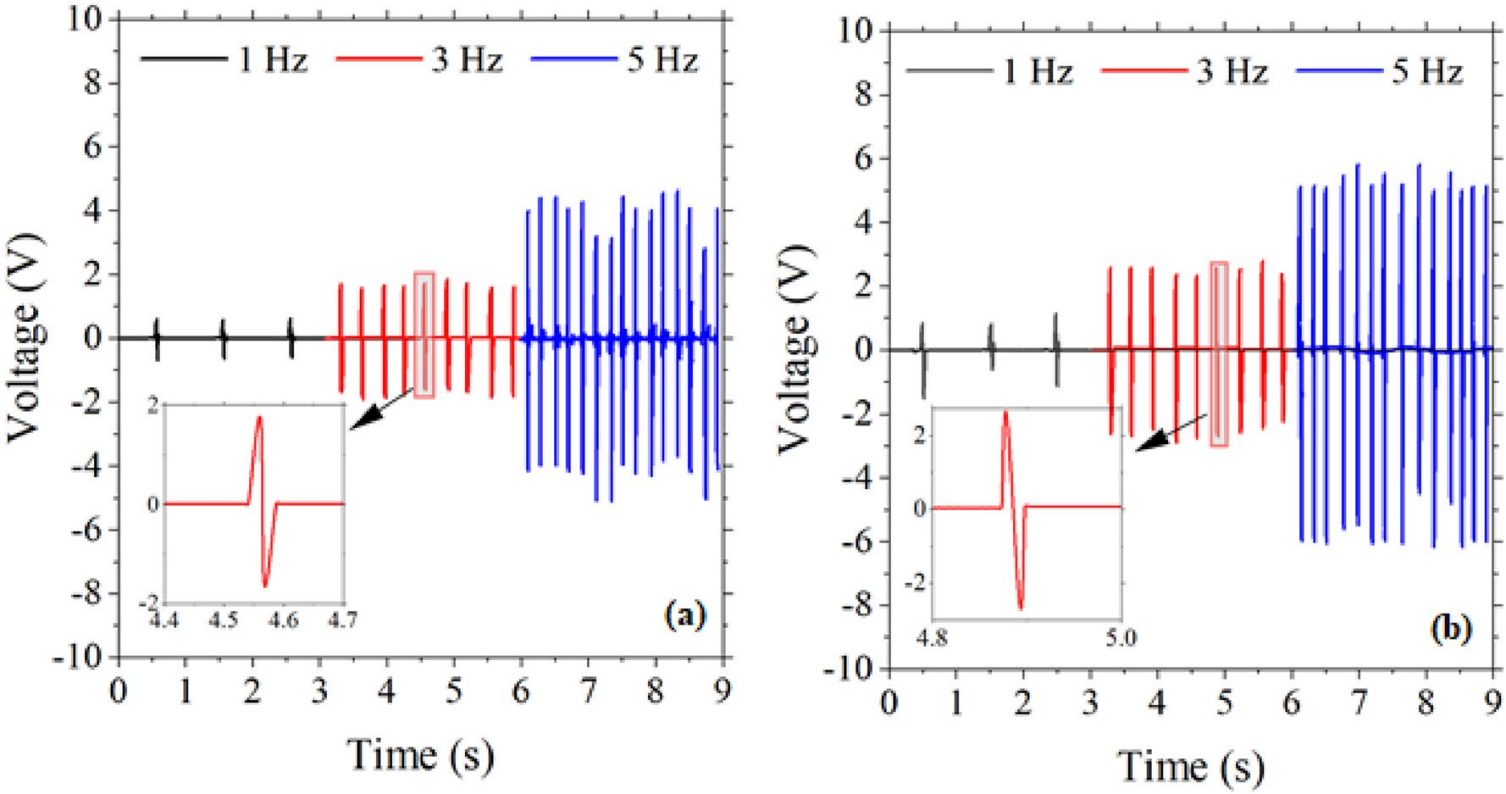
The output voltage of single-layer PENG based on (**a**) ZnO NRs (S-PENG1), (**b**) ZnO NSs (S-PENG2) (force 4 N). Insets are the zoomed view of output voltage.

Figure 4a,b shows the output voltages measured for S-PENG1 and S-PENG2, respectively, under the force of 4 N at frequencies of 1, 3 and 5 Hz. For both devices, the positive and negative peaks have been observed under compression and release owing to charging and discharging, respectively. As the frequency increased from 1 to 5 Hz, the average peak-to-peak voltage (Vpp) of 1.2, and 2.2 V, for S-PENG1 and S-PENG2, respectively, increased up to 8.1 and 11 V, i.e., the output voltage of both single-layer nanogenerator devices increased upon increasing the frequency. This enhancement in the output voltage with the applied frequency originates from the effect of the initial impact speed^33^. Insets of Fig. 4a,b show the magnified view of piezoelectric output voltage behavior.

It should be noted that the effect of frequency has been investigated only for single-layer PENGs. This is because a double-layer PENG, by itself, includes three parts that are different in their design and structure. Therefore, if the frequency would also be changing, the number of variable parameters would be larger than one, which precludes data comparison, and, consequently, the putative plots would not contain any useful information.

To investigate the effect of sandwich design on the performance of nanogenerator devices, the output of upper part, lower part and of the whole double-layer PENGs were measured and compared. The output voltage of the upper and lower parts of the PENGs refers to the measurements made between electrodes 1 and 2 and electrodes 2 and 3 depicted in Fig. 1f, respectively. For measurement of the total output voltage of the double-layer PENGs, the electrodes 1 and 3 were connected and the voltage was measured between electrodes 1 + 3 and 2.

**Figure 5:**
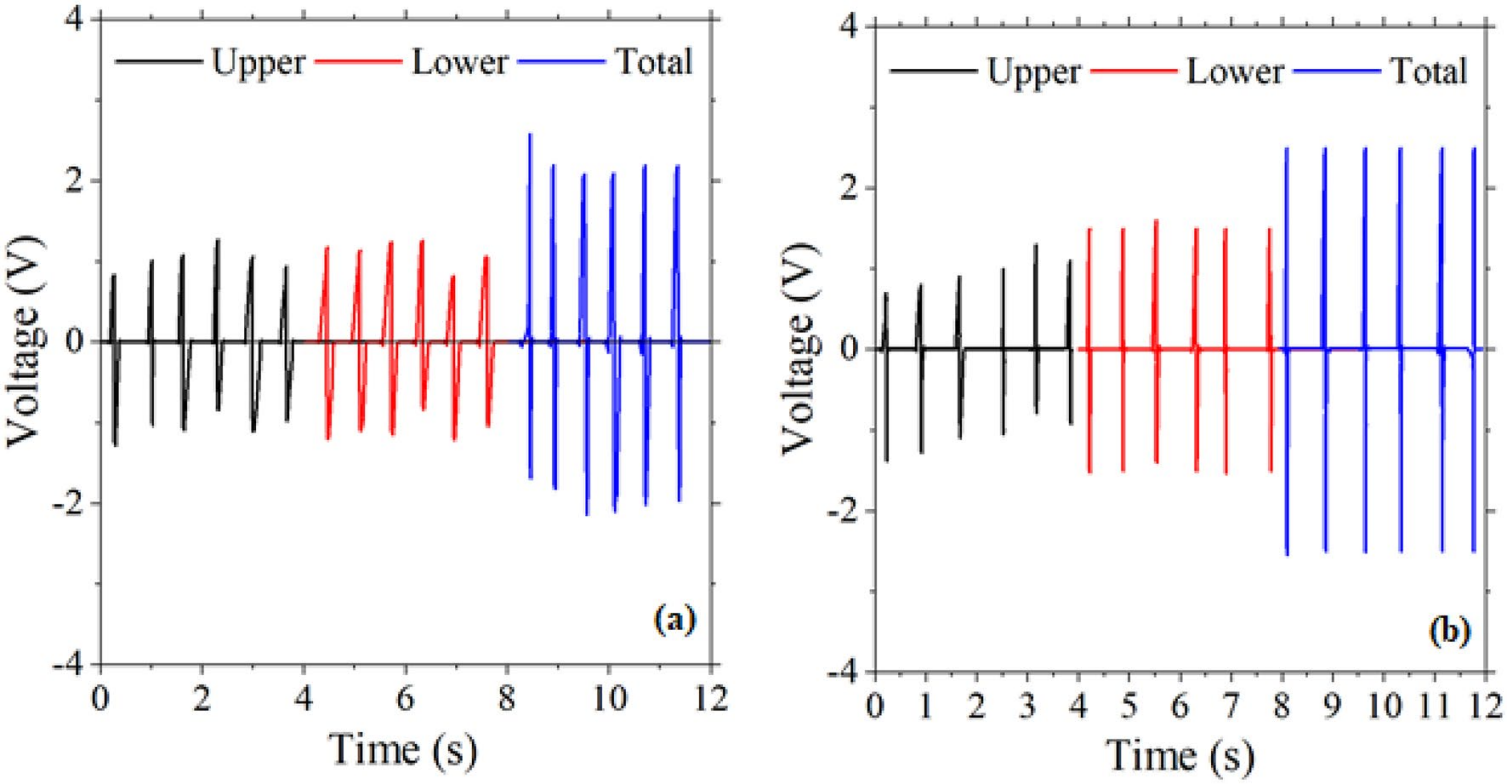
The output voltage of upper (up) and lower (down) parts and total output voltage of double-layer (**a**) D-PENG1 and (**b**) D-PENG2 fabricated devices (force: 4 N; frequency 1.5 Hz).

Figure 5 shows the upper, lower, and total output voltages of D-PENG1 and D-PENG2 under a force of 4 N and frequency of about 1.5 Hz. In the case of D-PENG1 (Fig. 5a), the upper and lower output voltages were found to be about 2 V, while a total voltage of 4 V was observed. The total output voltage of D-PENG1 has then been found to correspond to approximately the sum of the output voltages of the two parts (upper and bottom) of the device, each one being similar to that observed for S-PENG1. For D-PENG2 (Fig. 5b), the output voltages of the upper and lower parts were measured as 2.1 and 3 V, respectively, whereas the measured total output voltage was 5 V (i.e., also the sum of the output voltages of the two parts of the device). These results imply that the nanostructures act as synchronized charging pumps under periodic vertical impacts when compressed.

From the data shown in Fig. 5, the effect of the type of ZnO nanostructure on the output performance of nanogenerators can also be evaluated. For D-PENG1, the structure of the upper and lower parts are equal and are based on ZnO NRs, leading to identical output voltages (a result that indicates that, as could be anticipated, the two parts of the fabricated device work with a similar efficiency). On the other hand, D-PENG2 is a hybrid system composed of an upper part based on ZnO nanorods and a lower part based on ZnO nanosheets. The obtained results (Fig. 5b) demonstrated that the output voltage generated by the lower, ZnO nanosheets’ based part of the device was higher than that originated in the upper, ZnO nanorods’ based part. The better performance of nanosheets can be ascribed to their specific network structure along with the existence of vacant spaces (see Fig. 2c), which facilitate the transfer of the stress applied on a small area of the material to the entire network, thus leading to an effective deformation of a relatively large area upon mechanical stimuli. In addition, this result suggests also a comparatively higher surface polarization for the NSs (compared to NRs), which is compatible with an enhancement of the electromechanical coupling efficiency^34^.

**Figure 6:**
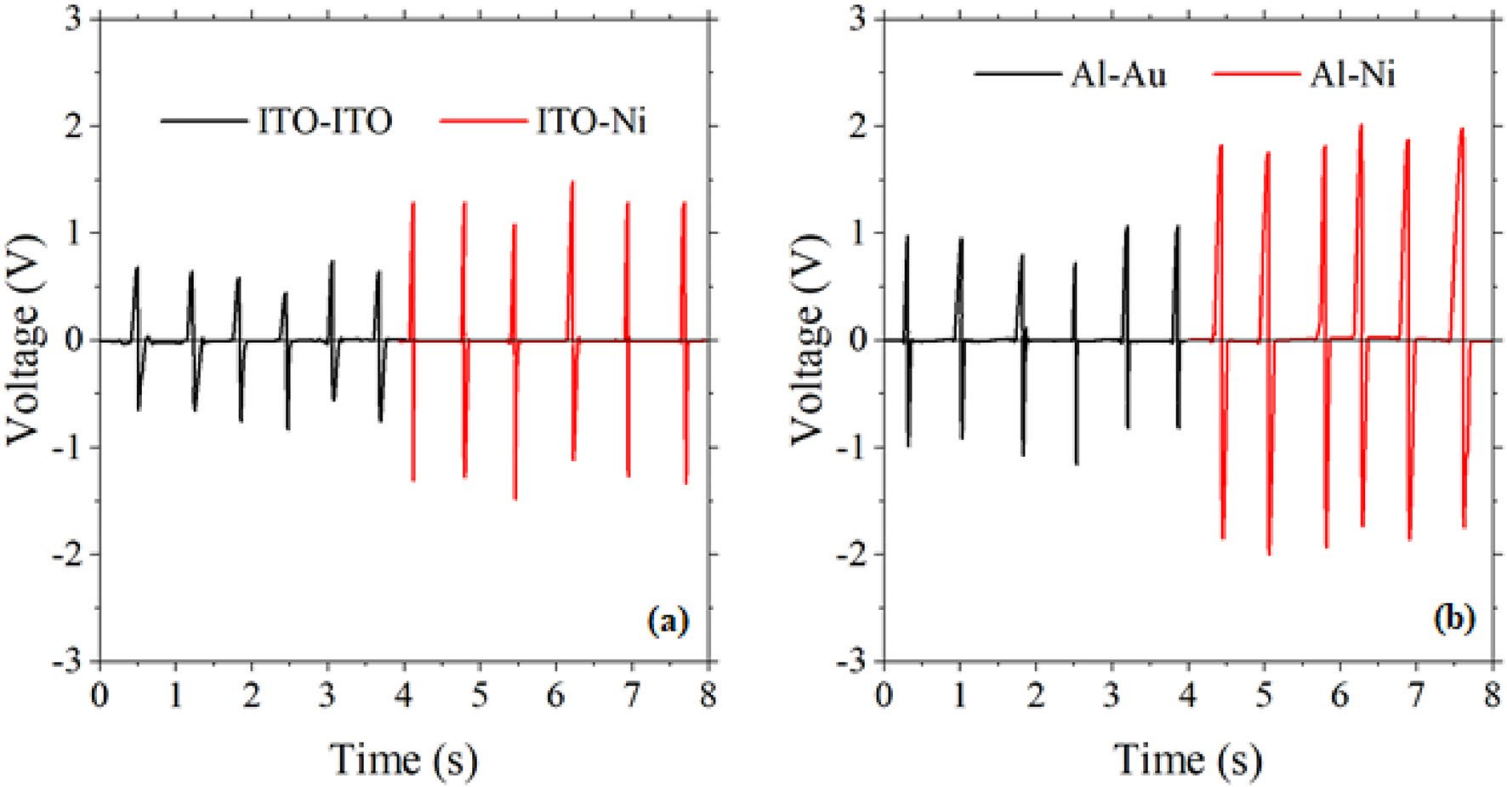
The output voltage of (**a**) D-PENG1-down (ITO-Ni) and S-PENG1 (ITO-ITO) and (**b**) D-PENG2-down (Al-Ni) to S-PENG2 (Al-Au) (force: 4 N; frequency 1.5 Hz).

Another parameter that can affect the output performance is the structure of the electrodes. In the PENG based on ZnO nanorods, four pairs of electrodes have been used including ITO-ITO, ITO-Ni, Al-Au, and Al-Ni, corresponding to S-PENG1, D-PENG1-down, S-PENG2 and D-PENG2-down, respectively. To investigate the effect of Ni compared to ITO, the output of D-PENG1-down (ITO-Ni) to S-PENG1 (ITO-ITO) under the same conditions should be compared. Also, the effect of Ni compared to Au, can be determined by comparing the output of D-PENG2-down (Al-Ni) to S-PENG2 (Al-Au). Figure 6a,b shows the output voltage of these four samples under the force of 4N at the frequency of 1.5 Hz. It can be concluded that the output performance of the ITO-Ni structure (about 2.4 V) is higher than that of the ITO-ITO (about 1.2 V). A similar result was also obtained from the comparison of Al-Ni and Al-Au, respectively, a result that may be attributed to the porous surface of the Ni foam, which has been shown to increase the output performance of PENGs^35^.

**Figure 7:**
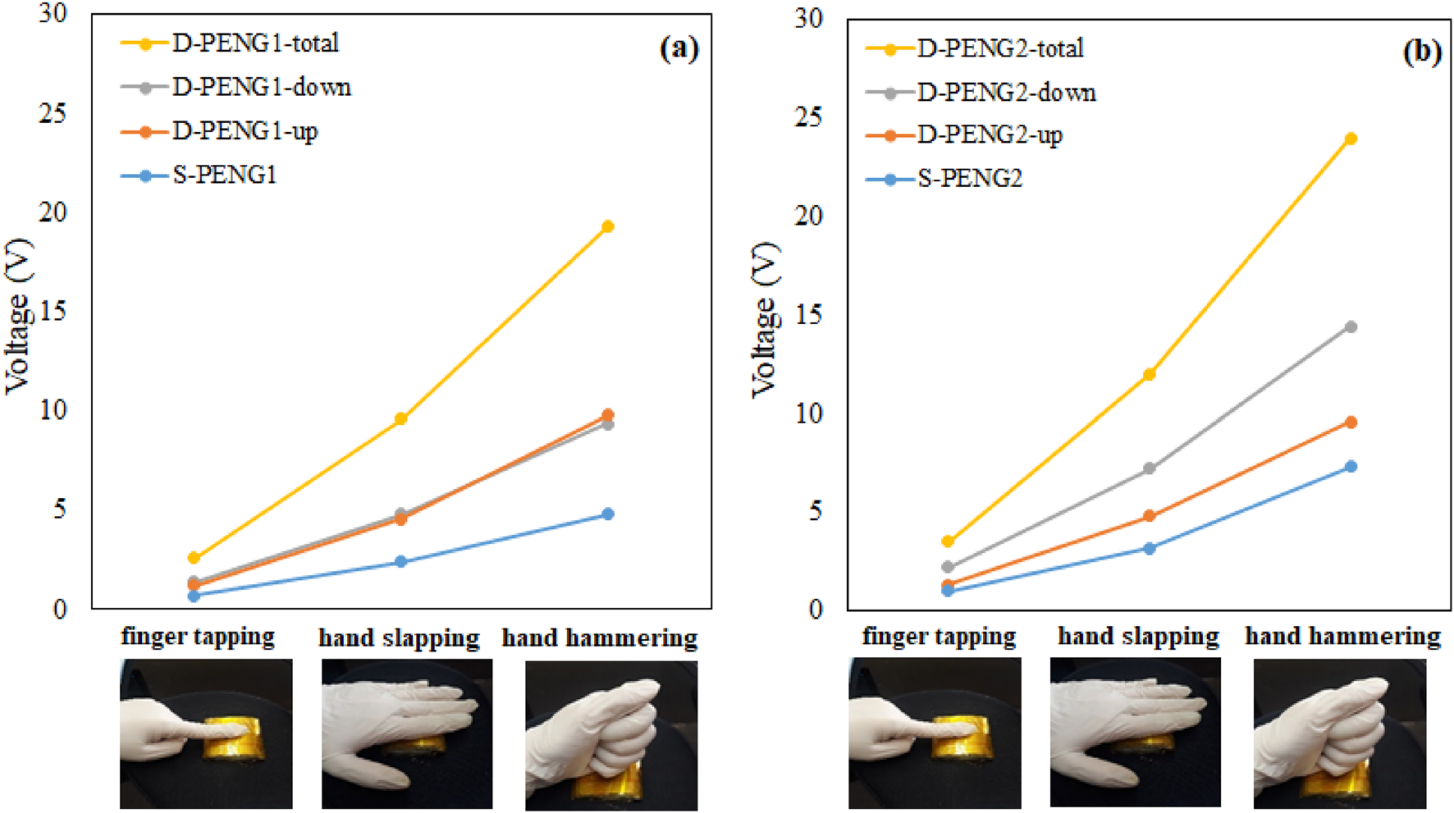
The output voltage of (**a**) PENG1 and (**b**) PENG2. Inset images show applying human impacts include finger tapping, hand slapping and hand hammering to measure the output of prepared nanogenerator devices.

To confirm the practical applicability of the prepared PENGs to convert the mechanical energy generated by human motions into electricity, the output voltages of all prepared nanogenerator devices were recorded under different human impacts, specifically finger tapping, hand slapping, and hand hammering. It should be noted that the force and frequency of the mechanical impact used to build Fig. 5 are also similar to human walking conditions. In fact, since the diameter of the used mechanical impactor was 9 mm, the applied pressure on the PENG devices was 62.9 kPa, which is similar to the applied pressure of a human foot during walking that, as explained in our previous work^36^, stays in the range of 60–80 kPa. The frequency of human walking is also about 1–2 Hz. Figure 7a,b shows the measured Vpp of single- and double-layer PENG1 and PENG2 devices under finger tapping, hand slapping and hand hammering. As it is shown in this figure, for all built PENG devices, the output voltage generated by hand hammering is clearly higher than those generated upon finger tapping and hand slapping. These results imply that the prepared PENGs can indeed be used as a sensor to detect human body activity. Such lightweight-flexible nanogenerators have also potential application in self-powered wearable electronic devices as multifunctional power sources.

The overall effect of various parameters discussed above (sandwich design, electrode structure, nanostructure of ZnO) on the piezoelectric output of PENGs can also be clearly seen in Fig. 7a,b. The porous structure of the Ni electrode can enhance the output about twofold, while ZnO nanosheets can result in an output voltage 1.5 times higher compared to ZnO nanorods, and use of a sandwich double-layer design can duplicate the output voltage of a single layer device. Overall, the piezoelectric output has been enhanced about five times for all applied forces when comparison is made between the performance of S-PENG1 and D-PENG2.

**Figure 8:**
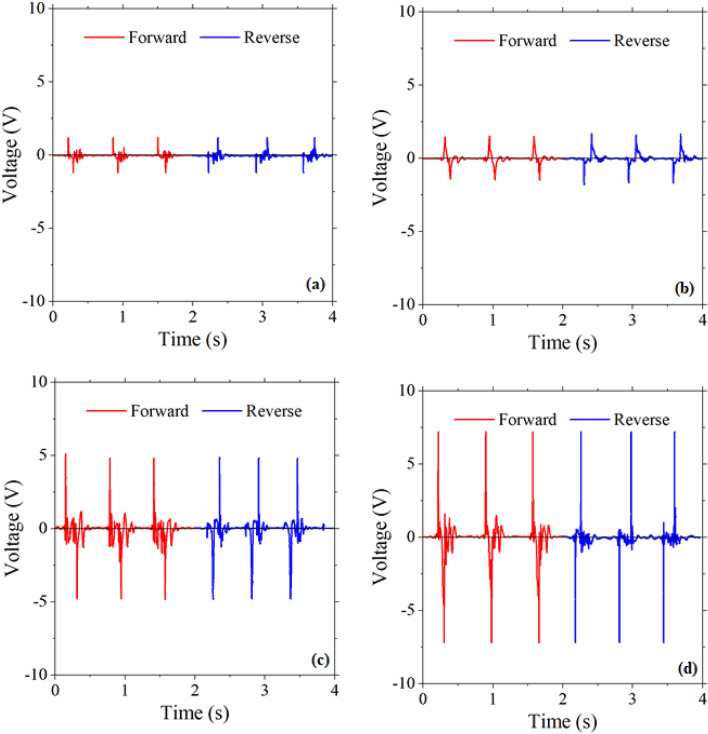
The piezoelectric output voltage of the PENGs at the forward and reverse connection for (**a**) S-PENG1, (**b**) S-PENG2, (**c**) D-PENG1-total and (**d**) D-PENG2-total (force: hand slapping, frequency: 1.5 Hz).

Figure 8 shows the piezoelectric output of prepared nanogenerator devices at the forward and reverse connection under hand slapping at the frequency of 1.5 Hz. Results show that the voltage peak was reversed by the device polarity switching, indicating that the piezoelectric signals originated from the nanogenerator and not from environmental noise.

**Figure 9:**
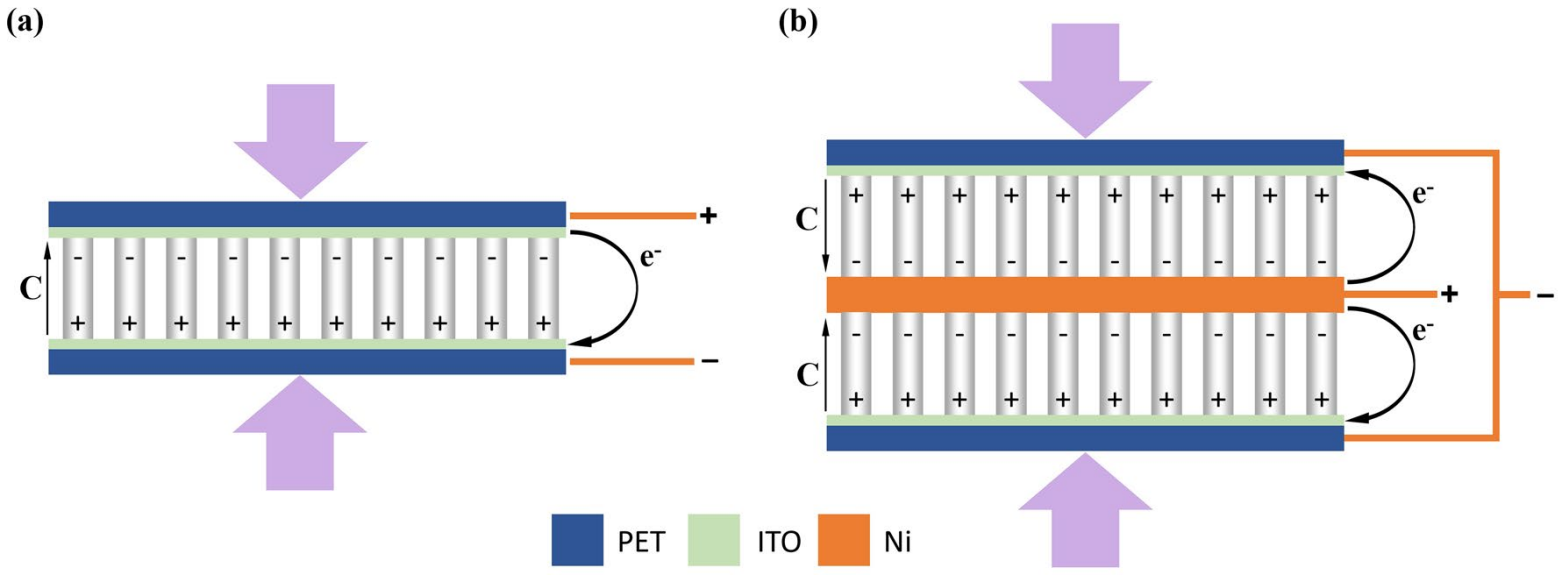
Working mechanism of (**a**) single-layer and (**b**) double-layer PENGs. The violet arrows represent the compressing stress force.

The working mechanism of single- and double-layer PENGs can be explained through the schematic diagram shown in Fig. 9. For a single-layer structure (Fig. 9a), when an external compressing stress is applied on a piezoelectric ZnO nanostructure, the reduction of the relative distance between O^2−^ anions and Zn^2+^ cations results in the generation of a piezoelectric field along the ZnO nanostructure growth direction (C axis in the figure), driving the electrons flow from the top electrode to the bottom one through the external circuit. On the other hand, in double-layer structures (Fig. 9b), the generated electric field enforces the electrons to flow from both sides of the PENG. These electrons gather together, resulting in the increment of the output voltage of the double-layer nanogenerator compared to the single-layer one. It should be noted that, although the piezoelectric output in a double-layer or even higher-layer PENG is higher than that of a single-layer PENG, however, the number of layers that can be used depends on the application for which the nanogenerator devices are designed. For example, for wearing applications, a large number of layers may negatively affect the comfortability and flexibility of the clothing. In fact, there are limitations in choosing the number of layers for each specific application and various factors to take into account in choosing the number of layers of a nanogenerator device for each application.

## Conclusion

In this work, we have demonstrated the novel double-layer piezoelectric nanogenerators based on 1-D (nanorods, grown on PET/ITO and PVDF/Au) and 2-D (nanosheets, grown on Al foil) ZnO nanostructures. Ni foam was used as middle electrode.

SEM and XRD results confirmed the growth of uniform and high-density, well-aligned vertically nanostructures.

The piezoelectric output of all prepared PENGs were evaluated under cyclic impacts at various forces and frequencies. The measured output voltage of the nanogenerators were compared in order to evaluate the effects of nanostructure type, structural design, electrodes structure and applied forces and frequencies value.

The results showed that the total output voltage in the double-layer PENGs is two times higher than those for the individual upper and lower parts of the device, while the use of Ni foam as middle electrode increased the output voltage by ca. twofold, compared to flat ITO and Au electrodes. Also, use of 2-D ZnO nanosheets for the construction of PENGs resulted in an enhanced output voltage by ca. 1.5 times when compared to that of devices where 1-D ZnO nanorods are employed.

Overall, it was found in this work that the design of nanogenerators in a double-layer sandwich structure based on 2-D ZnO nanosheets, with Ni foam as interlayer, brings relevant advantages as compared to ZnO-based single-layer and formerly proposed double-layer PENGs in terms of performance (higher output voltages; high flexibility), which increase the potential of this type of devices for practical applications. Moreover, both the overall preparation time of the devices and the number of required processing steps are convenient—reduce in comparison to two integrated single-layer devices.

## Data Availability

The data generated and analyzed during this study are available from the corresponding authors upon reasonable request.
